# International Comparisons of Pressure Standards: A Status Report

**DOI:** 10.6028/jres.093.146

**Published:** 1988-08-01

**Authors:** Charles R. Tilford

**Affiliations:** National Bureau of Standards Gaithersburg, MD 20899

**Keywords:** international comparison, pressure standards, primary standards, round robins, vacuum standards

## Abstract

In 1979 four working groups were established to organize comparisons between the pressure standards of the different national standards laboratories. These comparisons cover the range 10^−6^ to 10^8^ Pa. This report describes the progress of the different comparisons and summarizes the results where available.

## Introduction

In March 1979 representatives of 17 national standards laboratories met in Sevres, France under the auspices of the International Bureau of Weights and Measures (BIPM), to discuss pressure standards and metrology. A consensus was developed on several points: Pressure measurements are of major importance for both scientific endeavors and industrial enterprises; Although pressure is not one of the designated “base” units, it is in most cases maintained and promulgated in the same manner as the basic units; Comparisons of pressure standards between national laboratories have been very limited, and a systematic set of comparisons would be of great help in establishing confidence in the uncertainty analyses of the various national standards and in identifying problems; Progress is sometimes slowed because of the limited opportunities for pressure metrologists to meet and discuss problems and advances in the field, particularly in the medium pressure ranges.

As a result of these discussions four working groups were established, each with the primary task of organizing comparisons between the pressure standards maintained at national standards laboratories. The four working groups were: High Pressures (1 to 100 MPa) with G. F. Molinar of the Istituto de Metrologia “G. Colonnetti”, Turin, Italy, selected as Chairman; Medium or Barometric Pressures (1 to 1000 kPa) with P. R. Stuart of the National Physical Laboratory, Teddington, United Kingdom, as Chairman; Low Pressures (1 to 1000 Pa) with C. R. Tilford of the National Bureau of Standards, Gaithersburg, USA, as Chairman; and Very Low Pressures (10^−4^ to 1 Pa) with G. Messer of the Physikalisch-Technische Bundesanstalt, Berlin, German Federal Republic, as Chairman. The working groups separately met and began the tasks of identifying pilot laboratories, selecting transfer standards, and determining the number of intercomparison participants. Two years later, in June 1981, the International Conference on Weights and Measures (CIPM) established the Consultative Committee on Mass and Related Quantities (CCM), the related quantities being density, force, and pressure. The four pressure working groups established by the 1979 meeting were incorporated as CCM working groups with their activities to continue as previously planned.

The status of the intercomparisons organized by the four working groups are summarized below. It is of interest that in all four cases the intercomparisons have been delayed and/or compromised by problems with the transfer standards. In all but the medium pressure case larger-than-expected instabilities have been discovered, and in the low pressure and very low pressure cases these instabilities exceeded the uncertainties of the standards to be compared.

### High Pressures

The high pressure working group selected as an initial effort a comparison of pressure standards in the range 20 to 100 MPa. The Laboratoire National d’Essais (LNE), Paris, France, volunteered to act as the pilot laboratory. Two oil-operated simple piston gages were made available by a manufacturer for use as a transfer standard and backup. After characterization of the transfer gage and ancillary equipment, and selection. of a pressure fluid (diethylhexylsebacate), the comparisons were initiated in phases using the “petal” scheme, i.e., measurements of the transfer standard were made by the pilot laboratory at the beginning and end of each phase, or set up calibrations by a group of participating laboratories.

To date, three phases, including 13 laboratories, have been completed. The participants, in addition to LNE, were: First phase, Istituto di Metrologia “G. Colonnetti” (IMGC), Torino, Italy, Physikalisch-Technische Bundesanstalt (PTB), Braunschweig, German Federal Republic, National Physical Laboratory (NPL), Teddington, United Kingdom, and the National Bureau of Standards (NBS), Gaithersburg, USA. The second phase included Bundesamt für Eich-und Vermessungswesen (BEV), Vienna, Austria, Ceskoslovensky Metrologicky Ustav (CSMU), Bratislava, Czechslovakia, Aeronautical Research Institute (FFA), Bromma, Sweden, and Office Federal de Metrologie (EAM), Wabern, Switzerland. The third phase included the National Research Laboratory of Metrology (NRLM), Ibaraki, Japan, the National Institute of Metrology (NIM) Beijing, China, Amt für Standardisierung, Messwesen und Warenprufung (ASMU), Berlin, German Democratic Republic, and Gostandard-VNIIFTRI (VNIIFTRI), Moscow, Soviet Union. A fourth phase of the comparison is currently underway and will include the standards of Hungary, South Africa, Denmark, and India.

The procedures and results of the first three phases of this comparison have been detailed in separate publications by the participants, and an overall summary is presented in references [1] and [2], which include references to the earlier reports. In brief, each participant determined the effective area of the transfer standard (the gravitational force of the piston and weights divided by the applied pressure as determined by the participant’s standard) 17 times at 9 different pressures. The measured effective areas, *A* (*p*), corrected to 20°C, were then least-squares fitted to an equation of the form *A* (*p*)=*A*_0_ (1 + λ*p*). The zero-pressure effective area, *A*_0_, at 20°C, and the distortion coefficient, λ, were then used to characterize each participant’s results.

Repeated calibrations by the pilot laboratory of the transfer and backup gages indicated significant changes with time of the effective areas of the gages. It appears that the gages were asymptotically approaching a stable value, with the zero-pressure area of the transfer gage increasing from its initial value by 34 parts per million (ppm) over a 3-year period, and the backup gage’s effective area increasing by 52 ppm over a period of 4 years. In order to account for this, the effective areas of the transfer gage, as determined by the pilot laboratory, were least-squares fitted to a seven parameter equation with time and pressure as the variables. Residuals from this equation were no greater than 3 ppm. This equation was used to calculate pilot-laboratory values of *A*_0_ and λ appropriate to the time of each participants’ measurements.

The differences between each participant’s values of *A*_0_ and λ, and the appropriate pilot-laboratory values, were combined in a weighted average to generate reference values of *A*_0_ and λ. The inverse of the square of the uncertainty of each participant’s results were used as weighting factors. The uncertainties included the reported systematic uncertainties of the participants standards, and three times the standard deviations of the fitted values of *A*_0_ and λ obtained from the participant’s results. The reference values were refined in an iterative series of calculations by excluding from the average all results that differed from the refined reference values by more than the uncertainty of the participants’ results. In the end, the reference values for *A*_0_ and λ each included the weighted results from 9 of the 13 participants.

**Figure 1:**
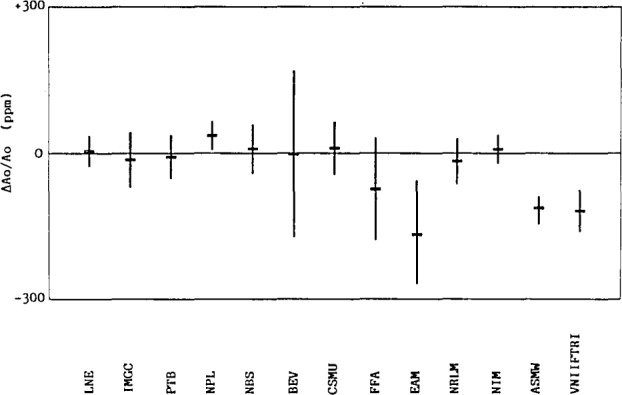
Fractional deviations of the zero pressure effective areas (*A*_0_) determined by each participant from a weighted mean reference value. The error bars represent the uncertainties of the measured differences, including the uncertainty of the participants’ standards, the uncertainty of the weights and thermometer used with the transfer standard, and three times the random uncertainty of the participants’ determinations of *A*_0_. Figure obtained, with permission, from reference [2].

Deviations between each participant’s results and the reference values of *A*_0_ are presented in reference [2] and similar results for λ and the effective area at 100 MPa (derived from *A*_0_ and λ) are in references [1] and [2]. The results for *A*_0_, including the uncertainty of each participants results, are shown in figure 1.

### Barometric Pressures

The barometric or medium range pressures working group decided that its initial effort would be a comparison of absolute pressure standards between 10 and 110 kPa. NPL, Teddington, volunteered to act as pilot laboratory. Gas-operated piston gages, operating in the absolute mode, were selected as the transfer standards. Two piston and cylinder sets were provided by NBS. A special piston gage base, with provision for changing of weights while under vacuum, was provided by the National Measurement Laboratory (NML), Sydney, Australia. A thermometer, vacuum gage and pressure control system were provided by NPL.

Initial characterization of the transfer gage by NPL indicated significant instabilities. These were traced to an anodized aluminum finish on the piston assembly. Desorption of water from this surface under vacuum conditions, and reabsorption of water when exposed to atmospheric air, were causing large mass changes. This problem was eliminated by stripping the anodization and replacing it with a nickel coating. Subsequent repeated calibration of the gage by NPL at each of 10 different pressures had a mean standard deviation of 0.08 Pa.

Eighteen different laboratories have expressed a desire to participate in this comparison. The first phase of this comparison involved NPL, BIPM, and the Institut National de Metrologie (INM), Paris, France. In all cases the primary standards were mercury manometers. Agreement between the three laboratories was ±0.5 Pa throughout the range, and none of the laboratories disagreed outside of their combined three sigma uncertainties.

Results are not yet available from a second phase, which will include CSMU, NML, and NBS. Further participation will be limited to laboratories that maintain independent primary standards, i.e., those whose standards are not traceable to the pressure standards of another laboratory.

The working group has proposed a further comparison of gage mode standards in the range 0.1 to 1 MPa. Eight laboratories have expressed an interest in participating in this comparison. However, no one has yet offered to act as pilot laboratory.

### Low Pressures

After an initial discussion of comparing low range differential pressure standards, the low pressure working group decided on a comparison of absolute pressure standards. NBS volunteered to act as the pilot laboratory, using a high resolution mercury manometer as the reference standard. Since the working group did not know of previous interlaboratory comparisons in this range, there was only limited experience to guide the selection of transfer standards. After discussing and evaluating several alternatives, the working group selected capacitance diaphragm gages. These electromechanical transducers have adequate precision for this pressure range and the laboratories likely to participate are familiar with their use since they are often calibrated for industrial calibration customers. However, their response in the lower part of the range is complicated by thermal transpiration effects, and their long-term stability had not been well established.

Four gages were available for the comparison, two provided by NBS and one each by two manufacturers. Two gages with a 10 Torr (1333 Pa) range were selected to provide coverage of the entire range of the comparison, and two with a 1 Torr (133 Pa) range to give better precision at the low end of the range. After initial calibration by the pilot laboratory, calibration by the first participant, NPL, Teddington, and recalibration by the pilot laboratory, it was evident that large shifts had occurred in some of the gages. Subsequent recahbration at the pilot laboratory showed further instabilities, even under laboratory conditions, with random magnitudes and direction. A second set of measurements was then made by NPL. The following calibration by the pilot laboratory showed smaller but still significant shifts in the gages. The decision was then made to suspend the comparison until the gage instabilities could be further evaluated.

**Figure 2:**
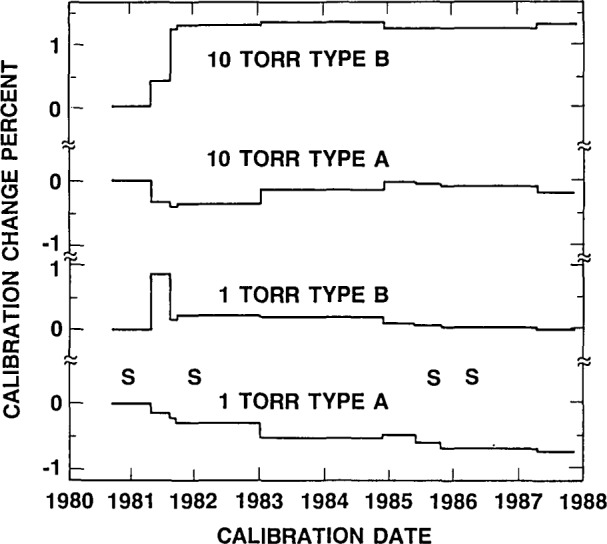
Changes with time of the calibration constants of the four transfer gages used for the low pressure comparison. The “S” marks the times when the gages were shipped overseas and back.

By this time data were becoming available on the recalibration of similar gages used by industrial standards laboratories. These data indicated large differences in the stability level of different gages. The instabilities observed for the transfer gages are consistent with the instabilities observed for the industrial gages [3]. The results of continued recalibration of the transfer gages are shown in figure 2 and indicate that the magnitude of the shifts have decreased with time, particularly for one type of gage (B).

The encouraging decrease in gage instabilities prompted a resumption of the comparisons. In 1985–87 measurements were made by PTB, Berlin and NML. Subsequent calibration by the pilot laboratory showed changes in the transfer gages that, while larger than desirable, were significantly reduced from the earlier experience. Results are currently being evaluated.

### Very Low Pressures

The very low pressure working group decided on a comparison with both argon and hydrogen. Argon is a widely used calibration gas, and hydrogen is an important gas for very low pressure metrology that presents special problems. PTB, Berlin, volunteered to be the pilot laboratory. The selection of a transfer gage was of some concern. Even under the best of circumstances, the widely used ionization gage is known to exhibit instabilities comparable to or larger than the uncertainties of primary standards. A possible alternative was a molecular drag gage. This gage measures pressures using the rotational decay rate of a magnetically suspended spinning ball (steel bearing ball) caused by collisions with gas molecules. This gage had only recently become available for routine laboratory use and only limited data were available on its stability, although these data were encouraging. In addition, the calibration of the gage is believed to depend only on the ball and the small gage tube containing the ball. Thus, only these small parts would have to be shipped to laboratories already possessing the gage electronics. Therefore, the decision was made to use the molecular drag gage; Kemforschungsanlage (KFA), Jülich, which was developing prototypes of the molecular drag gage for commercial production, made two electronic control units available for the intercomparison.

The working group developed a protocol involving a series of calibrations at specified pressures between 5 × 10^−4^ and 1 Pa. The pilot laboratory monitored the performance of the transfer gages before and after the measurements of each participant. For the initial participants, LNE, IMGC, NPL (Teddington), and CSMU, the transfer gages were hand-carried between laboratories, all of which were within a relatively short distance of the pilot laboratory. Measurements at the pilot laboratory indicated changes in the transfer gages of less than ±0.4% for any comparison. Larger changes were detected after measurements by the next two participants, NIM and NPL, New Delhi, India. In these cases the gages were also hand carried, but over much greater distances. Finally, after shipment by parcel post to and from NBS, the gages showed very large changes, as large as −8% for the hydrogen calibration of one gage.

The performance of the transfer gages and preliminary results of the comparisons, using argon, are detailed in reference [4]. It is believed that the cause of the instabilities is understood. The calibration factor of the molecular drag gage depends directly on the momentum transfer between gas molecules and the ball’s surface. It was believed that the surfaces could be stabilized by deliberately roughening them using an acid etch, and shipping the balls under vacuum in the stainless steel gage tubes. It now appears that the surfaces of the roughened balls were polished as they rolled around in the stainless steel tubes during transport, with increasing changes in the gage constant as the distance and violence of the transport increased.

**Figure 3:**
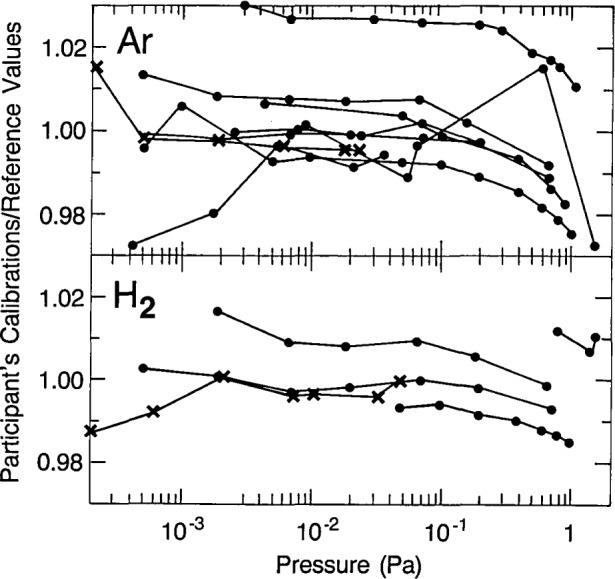
Preliminary results from seven of the participants in the very low pressure comparison, shown as dots, adapted from reference [4], with permission, with the second set of the results for NBS added as X’s. The values plotted are the average ratios, for the four transfer gages, of the gage calibration constant determined by the participants to the reference value determined by the pilot laboratory.

Subsequently, two new sets of four balls were characterized by the pilot laboratory and shipped to NBS, NPL (Teddington), and the Electro Technical Laboratory (ETL), Ibaraki, Japan. In this case the balls were restrained within the stainless steel tubes to minimize motion and the stability of the transfer gages have been satisfactory. The available calibration results for argon and hydrogen are shown in figure 3, and a final report is under discussion.
